# Efficacy of cineole in patients suffering from acute bronchitis: a placebo-controlled double-blind trial

**DOI:** 10.1186/1745-9974-9-25

**Published:** 2013-11-21

**Authors:** Juergen Fischer, Uwe Dethlefsen

**Affiliations:** 1 Norderney Hospital, Kaiserstr. 26, Norderney D-26548, Germany; 2 MKL Institute of Clinical Research, Pauwelsstr. 17, Aachen D-52074, Germany

**Keywords:** Acute bronchitis, Cough, Anti-inflammatory effects, Monoterpene Cineole

## Abstract

**Objective:**

Cineole has mucolytic, bronchodilating and anti-inflammatory properties and reduces the exacerbation rate in patients suffering from COPD, as well as ameliorates symptoms in patients suffering from asthma and rhinosinusitis. Based on these effects, we therefore postulated the hypothesis that patients with acute bronchitis would also benefit from therapy with Cineole.

**Methods:**

As part of a double-blind, placebo-controlled, multi-center-study, a total of 242 patients with confirmed acute bronchitis was randomly selected to participate. Over a period of 10 days, all patients were administered 3 x 200 mg of Cineole, or a respective placebo, per day. The primary outcome measure was a Bronchitis Sum Score, which summarises the relevant symptoms of acute bronchitis.

**Results:**

After 4 days of treatment it was notable, that the patient group treated with Cineole, showed significantly more improvements of the bronchitis-sum-score than those of the placebo group (p = 0.0383). The statistical significant difference of the individual outcome measures was especially underlined by the frequency of cough fits by p = 0.0001 after 4 days.

**Conclusions:**

The effects of Cineole in the treatment of acute bronchitis were clearly measurable and could be proven after a treatment period of merely 4 days. This study corroborates the fact that Cineole actively and significantly reduces cough frequency after four days. Therefore it has been shown to have a great socioeconomic impact.

**Trial registration:**

ISRCTN: ISRCTN37784439

## Introduction

Acute Bronchitis is one of the most common reasons for patient visits to ambulatory care or physicians. Frequently, it develops during the course of a common cold with a predominant symptom of dry or productive cough. On average, more than 50% of practice visits for acute bronchitis result in the prescription of antibiotics, although this is predominantly caused by viral infections. Evidence-based reviews and meta-analyses of randomized, controlled trials conclude that routine antibiotic treatment does not provide major clinical benefits in adults with acute bronchitis [1-7].

The primary goal should be the reduction of the frequency of cough fits, whereas treatment with antitussives is not recommended and should be reserved for specific exceptions. Mucolytic agents as aromatic essential oils, such as eucalyptus or peppermint oil, have a long history in treatment of respiratory inflammations. The main constituent of eucalyptus oil is Cineole, which has been proven as being effective in the treatment of respiratory diseases, such as rhinosinusitis, asthma and COPD [8-10]. This is essentially due to its mucolytic and primary anti-inflammatory effects. Therefore, the clinical effectiveness of Cineole has been repeatedly proven and established in the context of controlled clinical studies. As Cineole accelerates the beat frequency of the cilias in the mucous membrane, as well as acting both as a bronchodilating and an anti-inflammatory agent, it has to be postulated that it will be effective in treating symptoms of acute bronchitis. Therefore, in order to investigate the therapeutic effects of Cineole in the treatment of acute bronchitis, we conducted a randomized, placebo-controlled, multi-center study.

## Patients and methods

### Study design

This randomised, double-blind, placebo-controlled, parallel-group study was conducted in the practices of 3 general practitioners, 2 specialists in pneumology, 1 internal medicine and 1 ENT practice. In accordance with the Helsinki Declaration, the study was approved by the appropriate authorities and ethics committees, responsible for the 7 participating centers. Furthermore, all patients provided an explicit, written consent form, prior to their participation and the commencement of the study.

Patients were randomly assigned to one of the two treatment groups with stratification according to the clinical centers. Patients were then given the necessary dose of capsules, each containing either 200 mg Cineole, or no active ingredient. The dosage for each group amounted to 1 capsule, taken 3 times daily, resulting in a total dose of 600 mg of Cineole per day. The placebo control group received an equal dose of placebo capsules. In order to avoid patients from recognising the smell of Cineole, all patients were instructed to take the capsules with mineral water, half an hour before meals. All capsules, containing either the active substance, or the placebo, were organoleptically identical and sealed in blister stripes. All patients visited the practices at the beginning of the study, as well as in the subsequent 4 and 10 days.

### Enrolment of participants

The diagnosis of acute bronchitis was confirmed according to the criteria of Wenzel [7]. The study was limited to adult patients, aged 18 – 70 years, with a diagnosis of acute bronchitis not longer than 7 days. All eligible participants had a Bronchitis-Sum-Score of 7 or higher. Patients were excluded if they had severe medical conditions with relevant influence on the acute bronchitis.

### Outcome measures

As primary endpoint a Bronchitis-Sum-Score summarising relevant symptoms of acute bronchitis was defined as multiple criteria composed of the parameters dyspnea, sputum, frequency of cough, thoracic pain, auscultation and lung function – all equally weighted. These were specified for the intensity of dyspnea in scores as 0 = no difficulties in breathing, 1 = minor difficulties in breathing, 2 = moderate difficulties in breathing, 3 = severe difficulties in breathing and 4 = unbearable difficulties in breathing. Quantity of secretion was assessed by scores (0 = no secretion, 1 = < 2 ml (i.e. very modest), 2 = < 5 ml (i.e. modest), 3 = < 10 ml (i.e. moderate), 4 = > 10 ml (i.e. very distinctive). Frequency of coughing fits was documented according to patients diary with the scores 0 = no coughing fits per day, 1 = one coughing fit per day, 2 = 2 – 3 coughing fits per day (i.e. occasionally), 3 = 4–5 coughing fits per day, 4 = 4–9 coughing fits per day (i.e. frequent), 5 = > 15 coughing fits per day (unbearable often). Thoracic pain during coughing was measured by the scores 0 = no, 1 = modest, 2 = moderate, 3 = severe pain, 4 = unbearable pain. Findings of auscultation were measured by the scores 0 = no, 1 = modest, 2 = moderate, 3 = relatively distinctive, 4 = distinctive rales. Impairment of lung function was differentiated by the scores 0 = no, 1 = slight (90 – 99% of predicted value), 2 = moderate (80 – 90% of predicted value), 3 = strong (70 – 79% of predicted value) and 4 = very strong impairment (less than 70% of predicted value).

Additionally, symptom-scores were determined for dyspnea frequency and intensity during rest, as well as after exercise (scores: 0 = caused no problems, 1 = occasional problems, 2 = caused a lot of problems, 3 = the most important problem the patient had). The frequency of dyspnea during the course of a week was also gauged and converted into qualitative scores (scores: 0 = no day was good, 1 = 1–2 days were good, 2 = 3–4 days were good, 3 = nearly every day was good, 4 = every day was good). Additionally, coughing and the propensity to cough were determined, and given by scores (0 = no cough, 1 = in the morning without complaints, 2 = in the morning with complaints, 3 = in the morning and over the day (moderate), 3 = in the morning and over the day (severe), 5 = continuously during whole day (moderate complaints) and 6 = continuously during whole day (relevant complaints).

### Visits and randomisation

Randomisation was sequentially assigned in balanced blocks of 4 from a computer-generated list (random, idv Data-Analysis& Study Planning, Krailling, Germany). After the randomisation, the following patient details were recorded: height; weight; age; time from the first diagnosis of asthma symptoms; documentation of allergies; concomitant disease; prescribed medication; assessment of the current maintenance therapy. Control visits were carried out after 4 and 10 days, when adverse events were recorded and compliance with the treatment plan, as well as potential changes to therapy were addressed.

### Statistical analysis

The proposed sample size for this study was a total of 240 patients for both treatment groups (nnpar (nonparametric), idv, Data-Analysis & Study Planning, Krailling, Germany). An analysis of efficacy was performed with the intention-to-treat-population including all eligible patients, who received at least one dose of medication and had at least one follow-up visit. The primary outcome measure was composed by a Bronchitis-Sum-Score. The statistical analysis of the data was analysed using the Wilcoxon-Mann–Whitney-U Test, idv, Data-Analysis & Study Planning, Krailing, Germany). All secondary outcome measures were analysed using the Wilcoxon-Mann–Whitney-U Test as well. All data are expressed as mean values (with SD) and all tests were two-tailed. P-values of 0.05 or less were considered to indicate statistical significance.

## Results

A total of 242 patients – representing a real-life population - were randomised between February 2010 and January 2011 and received at least one dose of the study medication. Both treatment groups were well matched with respect to the baseline characteristics (Table 1). The mean age of the participants at entry was 41.0 and 43.9 years in the respective groups. The mean duration of the patients’ acute bronchitis was 3.9 and 4.0 days, respectively. The medication generally was not changed during the 10 days treatment period. The baseline parameters in both treatment groups were comparable. Treatment compliance was determined by counting the study medication at each visit and was found to be high and comparable across the treatment groups.

**Table 1:** Base line characteristics of the patients

**Characteristic**	**Placebo**	**Cineole**
	**(N = 121)**	**(N = 121)**
Age – yr		
Mean (SD)	43.9 (16.5)	41.0 (15.8)
Sex – M/F	54/67	50/71
Weight – kg		
Mean (SD)	76.3 (16.5)	77.8 (18.8)
Height – cm		
Mean (SD)	172.4 (9.1)	171.7 (9.0)
Days since detection of acute bronchitis		
Mean (SD)	4.0 (1.7)	3.9 (1.8)
Bronchitis-Sum-Score		
Mean (SD)	9.7 (2.5)	10.0 (2.0)
Smokers/exsmoker	35	32

### Primary outcome measures

Primary outcome measure was the Bronchitis-Sum-Score, composed of intensity of dyspnea; quantity of secretion, frequency of coughing fits; thoracic pain during coughing; rales according to auscultation; and impairment of lung function. After 4 days of treatment the mean decrease was 3.55 score-points in the Cineole group, and 2.91 score-points in the placebo-group (Table 2). The difference between both treatment groups was statistically significant after 4 days (p = 0.0383). Due to the amelioration of symptoms after seven to ten days without any medication, the difference between both treatment groups was found to be distinctive, yet not statistically significant at the 3rd control-visit after 10 days. At this point, the difference was calculated as 6.82 or 6.52. Comparisons of other single parameters of the Bronchitis-Sum-Score generally failed to show a statistically significant difference between both treatment groups, with the exception of the frequency of coughing fits.

**Table 2:** Change of primary outcome measures

	**Placebo**	**Cineole**	
**Mean of improvement**^**a**^	**mean**	**mean**	P-value*
Of intensity of dyspnea	0.42 (0.75)	0.44 (0.70)	0.8352
Of secretion quantity	0.42 (0.69)	0.44 (0.75)	0.8352
Of cough frequency	0.64 (0.87)	1.18 (1.12)	0.0001
Of thorax pain at coughing	0.58 (0.98)	0.60 (0.98)	0.5896
Of findings at auscultation	0.31 (1.17)	0.35 (1.15)	0.7198
Of lung function	0.42 (0.73)	0.34 (0.70)	0.3249
Of Sum Score	2.91 (2.803)	3.55 (3.022)	0.0383

Composed by the sum of symptoms after 4 days of treatment with cineole or placebo.

*P-values demonstrate the difference between the two treatment groups.

^a^Definition of the scores is defined in the paragraph with the outcome measures.

### Secondary outcome measures

#### Cough

The statistical significance of the individual parameters could also be proven, when the influence of treatment was compared on the basis of the frequency of coughing fits after 4 days (p = 0.0001). When measuring the cough documentation against a score generally used in other fields of research, the predominant parameter “cough” did not show a characteristic difference between both treatments (Table 3). Therefore, comparing coughing, or rather the cough frequency requires the correct definition, in order to show the different progress of symptoms.

**Table 3:** Comparison of amelioration of cough

	**Placebo**	**Cineole**	
	**Mean**	**Mean**	P- value^*^
**Frequency of coughing fits**^**a**^	0.64 (0.87)	1.18 (1.12)	0.0001
**Cough documentation**^**b**^	1.00 (1.39)	1.30 (1.31)	0.0896

Depending on definition of cough frequency and cough documentation. Change from baseline until 4 days of treatment with Cineole and placebo.

*P-values demonstrate the difference between the two treatment groups.

^a^Definition of the scores is specified for frequency of coughing fits: 0 = no coughing fits per day, 1 = one coughing fit per day, 2 = 2 – 3 coughing fits per day (i.e. occasionally), 3 = 4–5 coughing fits per day, 4 = 4–9 coughing fits per day i.e. frequent) , 5 = > 15 coughing fits per day (unbearable often).

^b^Definition of cough documentation is specified by scores: 0 = no cough, 1 = in the morning without complaints, 2 = in the morning with complaints, 3 = in the morning and over the day (moderate), 3 = in the morning and over the day (severe), 5 = continuously during whole day (moderate complaints) and 6 = continuously during whole day (relevant complaints) in the paragraph with the outcome measures.

### Side effects

A safety examination was carried out on all patients, who were administered the study medication. Within the placebo group, it was assumed that two of the recognised and recorded adverse events (e.g. gastrointestinal infection) were not related to the study medication, whereas a further AE was interpreted as being related to an intolerance of the study medication (i.e. heartburn and burning mouth). During treatment with Cineole 3 adverse events were reported as not being related to the study medication (otitis and sinusitis, eye burning, headache). In one case, a patient complained of stomach-aches, which was interpreted as being related to the study medication. It has to be noted, that the difference between the two treatment groups was neither clinically relevant, nor statistically significant. Safety examinations of the study and all participating patients highlighted no notable difference between the two treatment groups.

## Discussion

A number of controlled clinical trials on acute bronchitis have been carried out with view to the investigation of antibiotics. The results have shown, that antibiotics should be prescribed with care when other medications fail. Mucolytic remedies are traditionally used in Europe. A controlled clinical trial, based on Cineole as the main constituent of eucalyptus oil, had not yet been carried out on patients with acute bronchitis. In this trial, for the clinically relevant parameter of the reduction of cough frequency a statistically significant effect in favour of Cineole could be proven, due to the proven anti-inflammatory and mucolytic effects of this active ingredient.

Acute bronchitis is characterized by coughing that persists more than 5 days, while about 50% of patients produce purulent sputum that represents sloughed tracheobronchial epithelium and inflammatory cells. A rapid diagnostic test identifying a specific pathogen is normally not recommended. Most cases of acute bronchitis are caused by viral infections, where the major pathogens are influenza A and B viruses, parainfluenza virus, respiratory syncytial virus, corona virus, adenovirus, and rhinovirus. Knowing the pathophysiology it seems to be evident that antibiotics should not be the first choice of treatment. Therefore, treatment should focus on treatment options, which are known to have both favourable influence on the pathophysiology, and that are able to reduce the symptoms of acute bronchitis, without being limited by a relatively high incidence of side effects.

Mucociliary dysfunction has direct clinical implications on the pathophysiological mechanisms of acute bronchitis [11]. Based on the known anti-inflammatory and bronchodilating effects of the natural substance Cineole, it was assumed that this active ingredient is therapeutically beneficial for patients with acute bronchitis, thus, representing the necessary parameters of a useful and effective therapy.

An important point is the establishment of the correct points in time, at which, the parameters of progress of the symptoms are measured. This has to be viewed in the context of the duration of the existing diagnosis of acute bronchitis. This is a major concern in practise, because only a minority of patients will visit their physician immediately, following the recognition the first symptoms of an acute bronchitis. Therefore, we recruited the patients on the basis that their symptoms did not last longer than 7 days. The mean duration since occurrence of acute bronchitis symptoms was about four days. Thus, it is not surprising that the difference between the two groups, following 10 days of treatment with the study medication, no longer showed to be statistically significant. During the treatment period of 4 days the efficacy of Cineole compared with a placebo could be proven. The difference between both groups, based upon the symptom-sum-score and the frequency of coughing fits, decreased after 10 days, due to the normal course of symptoms without treatment of the disease. Considering the mean duration before beginning the treatment of the symptoms of acute bronchitis will also subside without treatment with an effective medication. Therefore, showing a statistically significant difference after 4 days of treatment has to be seen as a further contribution to the rapid onset of the efficacy of Cineole in treating acute bronchitis.

The interpretation of the results of this study corroborates the importance of selecting an effective Sum-Score. In our study, as in other investigations, we could clearly show that the number of coughing fits is the most relevant parameter to be chosen as demonstrating the progress of disease. The other parameters, in the acute bronchitis Sum-Score, were based on relevant symptoms of acute bronchitis, without showing equivalent improvement in comparison to the frequency of the coughing fits. The explanation for these finding, is that the baseline for these parameters requires a clear definition of the in- and exclusion criteria, when planning a study demonstrating the efficacy by these parameters. But as coughing is the main and predominant criterion for the definition of acute bronchitis, other parameters have only minor influence on the results.

## Conclusion

Cineole is effective in treating acute bronchitis, which can be explained by its proven pharmacodynamics and pharmacological properties. In other clinical studies the anti-inflammatory and also mucolytic effects could be proven in patients with Rhinosinusitis, COPD and Asthma. Our findings are complementary with these former results since the shown anti-tussive effects are also based on the amelioration of inflammation and of mucociliary clearance in patients with acute bronchitis.

Our results have to be viewed in the context of the fact that the disorder affects approximately 5% of adults annually. It belongs to the 10 most common illnesses among outpatients. The socio-economic relevance is further compounded by the short time frame in which relief of coughing and coughing fits are achieved, without the necessity of treatments with antibiotics. Amelioration of the main symptom frequency of coughing occurs without significant increase of costs in comparison to other treatment options.

## Competing interests

The authors declare that they have no competing interests.

## Authors’ contributions

The study was designed and the protocol developed by JF and UD. JF was the coordinating investigator. Statistical analysis was carried out by UD. The results were interpreted by JF and UD. Both authors gave substantial and critical input in writing and revising and approved the manuscript.
